# Anticancer peptides as novel immunomodulatory therapeutic candidates for cancer treatment

**DOI:** 10.37349/etat.2024.00264

**Published:** 2024-08-21

**Authors:** Apurva Sood, V.V. Jothiswaran, Amrita Singh, Anuradha Sharma

**Affiliations:** Changchun Institute of Applied Chemistry, Chinese Academy of Sciences, China; ^1^Department of Molecular Biology and Genetic Engineering, School of Bioengineering and Biosciences, Lovely Professional University, Punjab 144411, India; ^2^Biotechnology and Medical Engineering, National Institute of Technology, Rourkela 769005, India

**Keywords:** Anticancer peptides, cancer therapy, azurin, immunological warriors, cecropins, cancer

## Abstract

Cancer remains a concern after years of research in this field. Conventional therapies such as chemotherapy, radiation, and surgery are available for cancer treatment, but they are characterized by various side effects. There are several immunological challenges that make it difficult for the immune system and conventional therapies to treat cancer. Some of these challenges include heterogeneity, resistance to medicines, and cancer relapse. Even advanced treatments like immune checkpoint inhibitors (ICIs), which revolutionized cancer treatment, have associated toxicity and resistance further necessitate the exploration of alternative therapies. Anticancer peptides (ACPs) offer promising potential as cancer-fighting agents and address challenges such as treatment resistance, tumor heterogeneity, and metastasis. Although these peptides exist as components of the defense system in various plants, animals, fungi, etc., but can also be created synthetically and used as a new treatment measure. These peptides possess properties that make them appealing for cancer therapy, such as apoptosis induction, inhibition of angiogenesis, and cell membrane breakdown with low toxicity. Their capacity to specifically target cancer cells selectively holds promise for enhancing treatment environments as well as improving patients’ quality of life. This review provides detailed insights into the different prospects of ACPs, including their characterization, use as immunomodulatory agents in cancer treatment, and their mechanistic details after addressing various immunological challenges in existing cancer treatment strategies. In conclusion, ACPs have promising potential as novel cancer therapeutics due to their target specificity and fewer side effects than conventional therapies.

## Introduction

Cancer, which is still a complicated puzzle for researchers, affects millions of individuals despite several breakthroughs in therapy. Even after many years of screening, it continues to be the one of the major causes of fatality [1]. According to Global Cancer Observatory (GLOBOCAN) 2022 data, nearly 20.0 million new cases and approximately 9.7 million cancer deaths were reported globally [2]. The World Health Organization (WHO) reported 2.48 million lung cancer cases and 2.30 million breast cancer cases approximately in 2022. Further, colorectal cancer, prostate cancer, stomach cancer, liver cancer, cervix cancer, bladder cancer, non-Hodgkin lymphoma (NHL), and esophageal cancers come under the top ten list. Not only incidence wise but in terms of mortality caused lung cancer is the cancer with largest deaths followed by colorectal cancer. Breast cancer, stomach and liver cancer are also under the category with high mortality rate (if analyzed for both sex), however, the breast cancer is reported to be associated with highest incidence rate and mortality in females. Global estimates for new cancer cases are projected to reach approximately 35 million by 2050 [3].

Disease burden estimates are projected after consideration of several risk factors involving changes in population growth and aging. Socioeconomic development risk factors, including poor nutrition, a sedentary lifestyle, and polluted air, also contribute to the risk of cancer [4–6]. A thorough awareness of this illness is vital for developing cutting-edge treatment and prevention techniques to lower cancer incidence globally. These techniques not only significantly lower disease rates but also help to reduce healthcare and economic burdens [7–9].

The standard or conventional methods for treating cancer include multiple therapies, such as chemotherapy, radiotherapy, surgery, and hormone therapy. However, the effectiveness of such mainstream treatments is associated with various side effects that vary with the type of cancer, cancer stage, general state of the patient, and specific therapy protocol [10–12]. Usually, surgery is associated with pain, fatigue, bleeding, nausea, and a high chance of injury to adjacent organs/tissues [13]. Some side effects may occur immediately after chemotherapy, with nausea, vomiting, and anorexia being the most common [14]. Chemotherapy is also characterized by other side effects, such as fatigue, stomatitis, alopecia, hair loss, diarrhea, and peripheral neuropathies [15, 16]. Radiotherapy is another conventional therapy with side effects such as fatigue, hair loss (in the treated area), skin changes (redness, itching), and organ damage due to the use of high-energy radiation [17, 18].

Furthermore, side effects are even associated with recent treatments, including immunotherapy, hormone therapy, and transplants such as rashes, fever, diarrhea, and fatigue [19–21]. These may also cause inflammation of organs, resulting in unfavorable and fatal results [22]. Immune checkpoint blockade therapy has exhibited success in clinical trials, however, this therapy has been dominated by antibodies and associated with limitations like adverse immune reactions and limited responsiveness [23]. Bone marrow transplant complications can lead to infections, hemorrhage, and organ damage [24, 25]. Graft-versus-host disease is also likely to occur after allogeneic transplantation [26]. Another alternative for cancer treatment could be angiogenesis inhibitors, however, they are also associated with side effects such as proteinuria (excess protein in the urine), high blood pressure, bleeding and altered wound healing, thus greatly impact the quality of life of patients [27–29].

Another recent breakthrough in cancer treatment is chimeric antigen receptor (CAR) T-cell therapy. This involves the isolation of T cells from patients, which are further engineered to possess artificial receptors for specific tumor antigens [30, 31]. This seems to be a better treatment than other treatments, but it also has limitations. Some of these include critical toxicity, only fair antitumor activity, restricted trafficking, and limited tumor penetration [32]. CAR-T-cell therapy also requires a significant workforce and time to prepare engineered T cells. These limitations make this therapy slightly more complicated and questionable [33]. These shortcomings and adverse effects associated with already available therapies are also responsible for unaddressed gap between cancer diagnosis and treatment, hence warrants the development of new approaches to overcome these challenges.

Anticancer peptides (ACPs), also regarded as immunomodulatory agents, are assisting in the development of new means to overcome the constraints of conventional therapy. These potential treatment candidates are suggested to have more specific actions and are being targeted to develop new anticancer treatment measures with fewer side effects [34]. ACPs can surpass the body’s natural defenses and reach tumors effectively. These peptides can be used individually or in combination with other therapies like immunotherapy (immune checkpoint inhibitors) and chemotherapy [23, 35]. ACPs can also be designed as self-assembling peptides or in conjugation with nanocarriers which can further improve their delivery and reduce their breakdown by the enzymes of the body [36]. This review will discuss the immunological constraints in cancer treatments, evidence of different peptides exhibiting anticancer potential and their mechanism of action in detail. Further this article also discusses about the ACPs in clinical trials and the challenges associated with ACPs for enhanced translation.

## Methodology

A thorough literature search was carried out for the review paper using a number of internet sources. Initially, Google Scholar was used to obtain overall information about the current literature on ACPs, followed by a literature search using different databases, including PubMed, Google Scholar, and Scopus. The keywords used for the literature search were “anticancer peptides”, “immunomodulatory warriors”, “azurin and anticancer”, “cathelicidin and anticancer”, “magainin and anticancer”, etc.

The search was restricted to English-language papers that were published mainly in recent years (2000–2024). Based on their titles, abstracts, and complete texts, the retrieved articles were scrutinized for relevance. An extensive summary of the present state of research on peptides with anticancer activity and immunomodulatory features was then provided using the information retrieved from the chosen papers. Papers that were not related to anticancer or immunomodulatory activities of microbial peptides were excluded.

## Immunological issues in cancer and the need for ACPs

Cancer is typically characterized by immunological challenges that hinder the complete elimination of tumors from the body. Some of the main immunological issues are involved in the development of metastasis, relapse, heterogeneity, resistance to conventional drugs, and limited response rates to other therapies.

**Figure 1:**
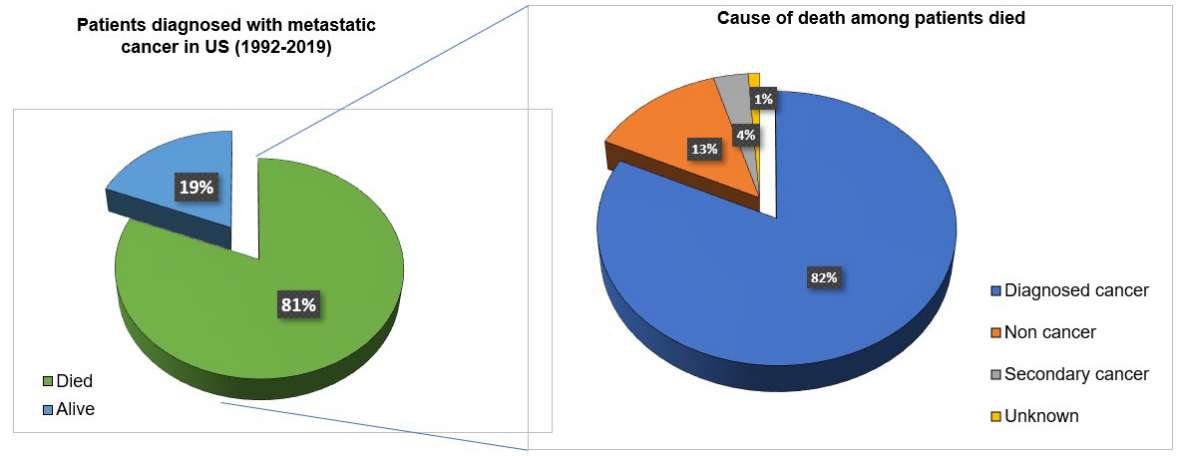
Metastatic cancer as a major cause of death in cancer patients. Out of 1,030,937 patients diagnosed with metastatic cancers in USA, 81% died during 1992–2019. Among the patients died the major cause of death was metastatic cancer (82.7%). 13% of the patients died due to non-cancer issues, 4% died due to secondary cancers and 1% were died due to reasons unknown

Metastasis or the dissemination of cancer cells from the primary tumor to distant organs is a major obstacle to successful treatment. A recent study published by Skomedal and his colleagues [37] mentioned that metalloproteinases play a significant role in the metastasis of cancer. The marine microalga *P. lutheri* has been shown to inhibit metalloproteinase-9 in HT1080 fibrosarcoma cells [37]. Metastasis leads to a maximum number of deaths related to cancer. Cancer metastasis involves the separation of cancer cells from the main tumor present at a specific site, invasion of the extracellular matrix and basement membrane, and eventual dissemination to other organs, usually through capillary blood [38]. This can make treatment difficult since newly metastasized cells may contain receptors different from those of the primary tumor. Receptor-targeted treatment can kill only primary tumor cells and not metastasized cells. Metastatic cancer is a major cause of death in cancer patients with a very less survival rate. A recent study referring SEER database reported 1,030,937 patients were diagnosed to have metastatic cancer in USA (1992–2019) out of which 81% (837,811) were found dead in follow ups. Among the died patients further the majority of patients i.e., 82.7% (688,529) died due to diagnosed metastatic cancer suggesting the implication of metastasis Figure 1 [39].

Another crucial immunological problem pertaining to cancer is heterogeneity, which is prevalent even within the tumor and its own metastasized or circulating form [40]. Breast cancer is one of the leading causes of death due to its heterogeneity [41]. For example, human epidermal growth factor receptor 2 (HER2) expression shows heterogeneity between primary tumors as well as the cells that have metastasized from them [42].

The relapse of cancer after treatment is another major risk factor linked to cancer [43]. A study by Kriegmair et al. [44] (2018) showed that relapse commonly occurs within 5 years of primary treatment. Some cancer cells, called cancer stem cells or stemness-high cancer cells, possess greater tumorigenic and metastatic activity than normal cancer cells. These cells are more likely to relapse within a short period of time [45]. The mainstream methods of cancer therapy have failed to address this issue, except for gene therapy, which has been shown to have some positive effects on cancer relapse [46].

Both inflammatory components and continuous exposure to antigens also pose serious issues leading to the depletion of T cells [47]. Cancer cells that have evaded immune surveillance can be eliminated by immunological warriors such as cytotoxic T cells and natural killer (NK) cells, which can recognize and kill cancer cells even if they have downregulated the expression of tumor-associated antigens or recruited immunosuppressive cells to the tumor microenvironment (TME) [15, 48, 49]. Patients can be treated with interleukin 2 (IL-2) and IL-15 to induce the production of NK cells, but this can lead to life-threatening toxicity and capillary leak syndrome [50]. Additionally, treatment with IL-2 can sometimes decrease the transport of NK cells to tumor-containing regions of the body [51].

Two major strategies used by cancer cells to escape immune system regulation include evasion from immune identification and creation of an immunosuppressive TME. In the first approach, cancer cells may undergo a reduction or loss of surface tumor antigenic expression, which protects these tumor cells from being recognized and targeted by cytotoxic T lymphocytes. For example, loss of human leukocyte antigens (HLAs) in non-small cell lung cancers leads to immune escape through the presentation of fewer antigens [52]. Additionally, mutations and deletions may result in downregulation of the antigen-presenting machinery, conferring resistance to T-cell effector genes. As genomic tumor heterogeneity increases, the probability of sub clonal generations escaping immune attack also increases. Metastasis progression and therapeutic resistance usually occur due to the presence of rare clones in primary tumors. Furthermore, cancer cells evolve mechanisms that mimic peripheral tolerance, preventing the local cytotoxic response of effector T cells, as well as that of other cells, such as tumor-associated macrophages (TAMs), NK cells, and tumor-associated neutrophils (TANs) [52].

**Figure 2:**
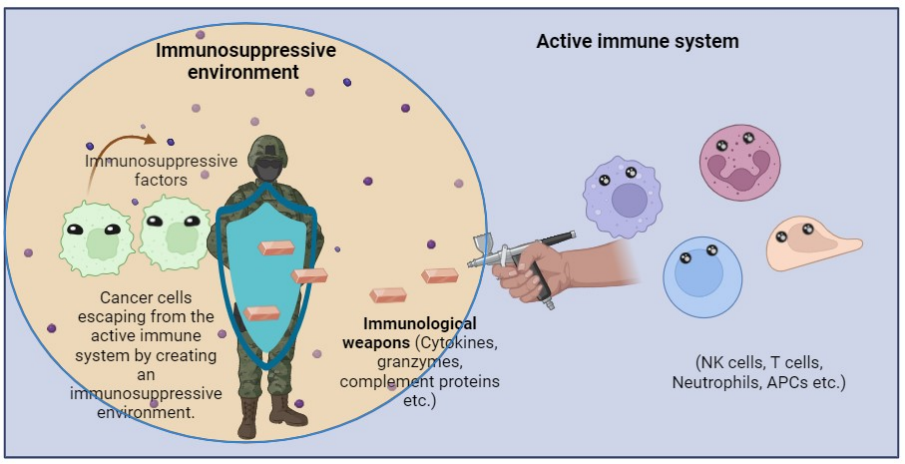
Cancer cells escaping from the immune cells in an immunosuppressive environment. The immunosuppressive factors make cancer cells evade immune detection and affect macrophage function, promoting the growth of tumor and suppressing immune cell response. APCs: antigen-presenting cells. Created with BioRender

In the second strategy, cancer cell-derived factors instigate an immune-tolerant TME by secreting suppressive molecules, expressing inhibitory checkpoint molecules, and inducing the recruitment of immunosuppressive cells such as TAMs, myeloid-derived suppressor cells (MDSCs), and regulatory T cells (Tregs). These strategies result in a complex and efficient system for immune evasion. Therefore, cancer cells avoid destruction by immune attack through a combination of strategies that prevent immune recognition and create an immunosuppressive microenvironment as shown in Figure 2 [52].

**Figure 3:**
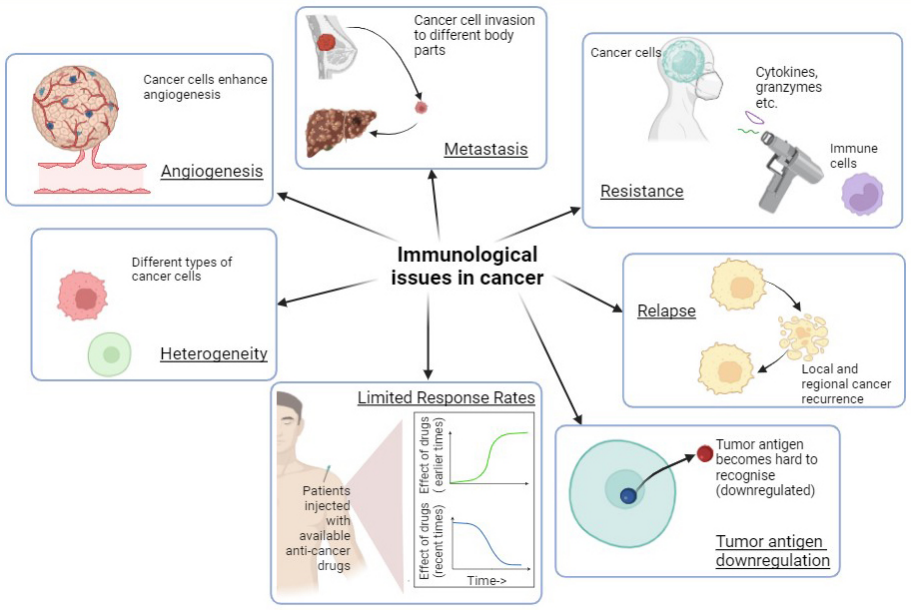
Various immunological challenges in cancer render treatment ineffective and warrant the development of immunological warriors as new treatment strategies. Created with BioRender

A limited response rate is another major issue that is an obstacle during the treatment of cancer. Tumors have revolutionized current cancer treatments, leading to low response rates [53]. Due to primary and secondary resistance, single agent immunotherapy frequently fails, and only a small percentage of patients have lasting advantages promoting the use of combination therapies. With time, even combination therapy is losing its effect due to tumor mutations, leading to the demand for newer therapies for cancer treatment [54]. The various immunological challenges have been summarized in the Figure 3.

ACPs are short peptides that can specifically bind to tumor-associated cells (TAAs) and activate cytotoxic T cells and NK cells, leading to the killing of cancer cells, even those that have evaded the immune system. Cancer cell resistance to various existing therapies also demands the formulation of new ways to combat cancer [55]. Some malignancies become resistant to inhibitors and other immunotherapies. Immunotherapies can be used in combination with immunological warriors. This improves the effectiveness of therapy and aids in overcoming resistance [56].

Tumors require blood vessels to grow, which is another issue related to cancer. There are certain tumors that control angiogenesis to survive [57]. ACPs may target the vessels that lead to tumors to reduce the nutrient supply and access growth factors. This ultimately results in the fostering of anticancer immunity [58]. Persistent inflammation in the TME is another factor that promotes tumor growth. The activation of ACPs and immune cells limits the inflammatory response to control cancer growth by managing inflammation [59, 60]. Immunological stressors can establish immune memory. They can function by identifying and killing remaining cancer cells. This leads to long-term protection against tumor recurrence [61].

## Immunomodulatory agents and their classification

ACPs are commonly known as immunomodulatory agents due to their ability to generate a strong immune response and help the immune system fight tumor cells. These peptides are usually between 10 and 60 amino acids in length [62].

There are different ACPs that can be classified according to various parameters. Some of these classifications are according to origin, structure, mechanism of action, and target specificity. In terms of origin, these peptides can be obtained from plants, insects, microbes and mammals, where they serve as a part of the pathogen response system [63]. For example, Alfer is an anticancer microbial peptide obtained from insects that has been demonstrated to be effective against human papillomavirus [64]. Thiocoraline is a novel peptide of microbial origin (obtained from the actinomycete *Micromonospora marina*) with anticancer activity [65]. Bacteria also produce peptide bacteriocins that have been shown to have anticancer effects [66]. The cathelicidin family include peptides of mammalian origin. Additionally, synthetically designed peptides such as acyl-lysine oligomers are also reported to possess inhibiting properties [67].

Based on their structure, anticancer microbial peptides can be classified as either α-helices, β-sheets, random coils or cyclic peptides [64, 68]. ACPs can also be classified on the basis of other characteristics like mechanism of action, target specificity and origin. On the basis of their mechanisms of action ACPs can be classified as membrane disrupters, immune regulators, angiogenesis inhibitors, and apoptosis inducers [62, 69]. The different ACP classifications are summarized in Table 1.

**Table 1:** Classification of anticancer peptides with their examples

**Classification**	**Subsections**	**Examples**
Origin	Bacterial	E.g., Nisin [70]
	Plant-based	E.g., Cyclotides [71]
	Fungal	E.g., Alamethicin [72]
	Animal origin (Mammals)	E.g., Cathelicidins [73]
	Synthetic	E.g., PR-35 [62]
Structure	α-Helical	E.g., Aurein [74]
	β-Sheet	E.g., LfcinB-P13 [75]
	Random coil	E.g., KW-WK/Alloferon [62]
	Cyclic	E.g., Diffusa Cytide 1 [62]
Mechanism of action	Pore formation	E.g., Pardaxin [76]
	Immune regulators	E.g., LfcinB [75]
	Angiogenesis inhibitors	E.g., Temporin-1CEa [62]
	Apoptosis inducers	E.g., Dolastine 10 [77]
Target specificity	Specific	E.g., Cecropins [78]
	Broad spectrum	E.g., Defensins [79]

They are also subdivided into two classes according to target specificity. The first category includes ACPs directed toward specific target cells without affecting healthy cells. A common example of such a type includes cecropins, whereas the second category comprises broad spectrum ACPs that act on various tumor types as well as normal cells. HNP-1 to 3 (human neutrophil defensins 1-3) are examples of broad-spectrum ACPs [68]. The knowledge of their classification is helpful in choosing various types of ACPs depending upon the cancer type, which leads to higher efficacy and greater success during clinical trials.

These peptides are being researched as potential treatments for different types of cancer because their precise tumor-targeting properties have been demonstrated in numerous trials. They also constitute an effective substitute for routine chemotherapy [80]. Some antimicrobial peptides with anticancer properties include magainin, nisin, and cecropins. Researchers have expressed interest in using these peptides for cancer treatment for many years. However, the efficacy and safety of these peptides have still not been demonstrated in clinical settings.

## Anticancer potential of some major ACPs

### Azurin

Azurin is a minor protein that is secreted by *Pseudomonas aeruginosa* [81]. It is a 128 amino acid long copper-containing peptide that belongs to the cupredoxin family [82]. It enters cancer cells and prevents them from growing and proliferating. Research has revealed that it functions as an antitumor agent by blocking angiogenesis. This process is carried out by phosphorylating vascular endothelial growth factor receptor 2 (VEGFR2) [83]. Research has also revealed that the cytosolic dual-specificity phosphatase 1 (COP1) protein leads to ubiquitination of p53, which causes its degradation, but the p28 fragment of azurin binds to p53 and disrupts the binding of COP1 to p53. This leads to the stabilization of p53, which eventually leads to apoptosis [84]. Azurin, when injected into mice with human cancer cells, has shown success in tumor regression [85].

Azurin is also known to alter cancer cell surface structure by affecting the specific lipid caveolae or rafts present in the cell membrane. These microdomains are targeted by azurin resulting in accelerated internalization of overexpressed surface receptors and inactivation of hyperactivated signaling pathways specific to cancer cells [86]. Different domains of Azurin play different roles such as p28 domain facilitate its entrance into cancer cells, whereas, C domain exhibit the anticancer properties by interfering with the signaling required for cell growth [86]. Azurin has the ability to leave normal cells unaffected by targeting cancer cells and has also been demonstrated to have fewer side effects [87].

A study by Ramachandran and Mandal showed that when mice were treated with azurin, the lifespan increased by 94.19% [87]. Furthermore, another interesting study demonstrated the inhibitory potential of azurin against metastasis, where recombinant azurin (expressed in *E. coli* Nissle 1917) inhibited metastasis in immunocompetent mice with implanted 4T1 breast cancer. This study used the tissue targeting property of *E. coli* to suppress the growth of 4T1 breast cancer and prevent metastasis in mice [88]. A study by Karpiński and Adamczak also demonstrated apoptosis in J774 cells from a reticulum cell sarcoma. Azurin increases the intracellular concentration of p53 in the nucleus, which causes the release of cytochrome c into the cytosol, ultimately leading to apoptosis [82]. Another study demonstrated that azurin-treated YD9 cells had lower viability than usual cells. Azurin at 200 µg/mL slowed cell proliferation by approximately 50% [89].

A recent review suggested that azurin-P28 enters endothelial cells, interacts with p53 and promotes its expression, which further leads to cell death as cell growth is halted at the G2/M stage [83]. Further, azurin immobilized with nano chitosan exhibited anti-cancer properties on various cancer cell lines, such as CLS-145 (gastric), AsPC-1 (pancreatic) and HepG2 (liver) [90]. Azurin has also been noted to interfere with glioblastoma cells by forming fusion proteins with *Neisseria meningitidis*’ azurin-like protein Laz’s N-terminus (H.8 moiety). Azurin alone cannot enter glioblastoma cells, but this fusion product can enter cells and shows increased cytotoxicity, leading to disruption of the entry barrier [91]. Thus, the potential of azurin as an ACP is promising.

### Lactaptin

Lactaptin, a kappa-casein proteolytic fragment of human breast milk, has been demonstrated to have anticancer properties [92]. It promotes the synthesis of cytokines and chemokines by the immune system, which targets cancer cells [93]. Further, reports have indicated that lactaptin is more specific and more effective at targeting cancer cells while leaving normal cells unaffected, unlike chemotherapy, which destroys normal cells alongside cancerous organs [94].

Lactaptin’s recombinant analog RL-2 has been reported to induce the death of cancer cells in preclinical studies [95]. It causes apoptosis in cancer cells by interacting with surface receptors on cancer cells and triggering the initiation of an immunogenic cascade exhibiting anticancer properties [96]. This strengthens the immune system and improves its capacity to identify and combat cancerous cells. Apart from its impact on cancerous cells, lactaptin was found to decrease swelling, which promoted wound healing. RL-2 was shown to stimulate MCF-7 cancer cells to undergo apoptosis [97]. It is responsible for the induction of caspases, along with the loss of mitochondrial membrane potential other than phosphatidyl externalization, in MCF-7 cells and hence apoptosis [92]. Koval et al. [98] (2014) showed that lactaptin effectively inhibited the growth of breast cancer cells (MDA-MB-231) as well as solid tumors induced as xenografts in mice without causing severe side effects. The RL-2 peptide upregulated the expression of apoptosis-related genes, including caspase-3 and 7, and suppressed the expression of antiapoptotic genes, such as Bcl-2, suggesting the induction of apoptosis. Furthermore, this peptide also induced the expression of LC3 (microtubule-associated protein 1 light chain 3), which is a hallmark of autophagy. RL-2 also induces several alterations, such as the externalization of phosphatidylserine (PS) and altered mitochondrial membrane potential in hepatocarcinoma A1 cells *in vitro*, mainly concerning the dissipation of membrane potential in mitochondria and the translocation of PS to the outer cell [98]. The same study demonstrated an antitumor effect of RL-2 when it was administered with cyclophosphamide. Individually, RL-2 for solid tumors showed an inhibitory effect at a therapeutic dose of 125 mg/kg, which was comparable to that of cyclophosphamide alone at a therapeutic dose of 150 mg/kg.

Anticancer drugs are potent enough to induce autophagy, and in similar lines, RL-2 promoted autophagosome and reactive oxygen species accumulation. Hence, autophagy inhibitors such as 3-methyladenine, chloroquine and Ku55933, along with RL-2, can enhance the cytotoxic effect on cancer cells. The efficacy of traditional chemotherapy drugs can also be enhanced by the use of lactaptin in combination with other drugs, as shown in another study [99]. This finding suggests that lactaptin could be an effective supplement to traditional cancer therapies. As studies on lactaptin are progressing, scientists are looking for alternative means of using this potent peptide.

Lactaptin has also been shown to suppress innate immune reactions by inhibiting NF-κB signaling. TNF-alpha-induced protein 3 (A20), inhibitor of apoptosis (IAP), and cellular FLICE-inhibitory protein (cFLIP) are target genes for the NF-κB cascade rather than the cellular immune system and hence promote cancer cell apoptosis. RL-2 weakly evokes an immunogenic response in immunocompromised mice as well as in adenocarcinoma of the breast, suggesting that it is a weak immunogen and a possible candidate for molecular treatment [97, 100]. In conclusion, lactaptin has the potential to induce an immune response and could be a promising candidate for anticancer drug development. Preliminary research seems quite promising, but further investigation of the optimal dose and treatment options for other delivery methods is still needed to improve the success of these agents as anticancer therapeutics.

### Cathelicidin

Cathelicidins, which are amphiphilic peptides with 12–97 amino acids, were first discovered in bovine neutrophils by Zanetti and collaborators in the early 1990s [101]. The human body has various types of immune cells that can secrete antimicrobial peptides. Cathelicidin is one of the major peptides synthesized by innate immune cells and cells that are in contact with the environment, such as mucosal epithelial cells, circulating phagocytic cells and keratinocytes [73]. It can fold into amphipathic α-helices as a secondary structure. This molecule has a conserved domain at the N-terminus called cathelin and a variable carboxyl-terminal sequence [67].

In humans, only one cathelicidin has been discovered in the myeloid bone marrow, which preferentially disrupts bacterial and cancer cell membranes rather than host eukaryotic cell membranes due to the presence of a large amount of negatively charged PS on cancer cells compared with that on normal eukaryotic cells [102]. A study reported *Mycobacterium tuberculosis*-based induction of cathelicidin production in various cells, such as neutrophils, epithelial cells, and alveolar macrophages, which accounted for the activation of Toll-like receptors [103]. Intestinal bacteria are responsible for producing metabolites such as short fatty acid chains and butyrate, which act as a force to induce LL37 production [104]. *Helicobacter pylori* infection, as reported previously, induces the expression of the mature form of human cathelicidin LL-37 during the early stage of infection [105].

Scratch and colony formation assays were performed on human U251 glioma cells treated with the human cathelicidin LL-37, which exhibited an inhibitory effect on the migration and clonogenicity of glioma cells. The overexpression of epidermal growth factor receptor de2-7EGFR or EGFRvIII and moderator expression in the mitochondria inhibits the dependency on glucose and enhances oxidative metabolism. A Mito Stress Test Assay on LL-37-treated glioma cells revealed that the respiratory capacity of mitochondria was reduced by inhibiting ATP production in human glioma cells [106]. The overexpression of anionic components on the surface of cancer cells, such as glycolipids, glycoproteins, proteoglycans, etc., increases the vulnerability of cancer cells to cationic antimicrobial peptides. One study demonstrated that the cathelicidin-related peptide FF/CAP18 has antiproliferative effects on two major cancer cell lines, oral squamous SAS-H1 and colon cancer HCT-116 [107, 108]. 17BIPHE2 derived from LL-37 is the shortest antibacterial peptide which has been shown to cause high levels of apoptosis in A549 lung cancer cells with a high amount of ROS generation, probably due to high levels of extracellular signal-regulated kinase (ERK) phosphorylation and Bcl-2-associated protein x (BAX) expression [109].

In humans, cathelicidin, called CAMP, has been shown to have a double-sided effect. It kills cancer cells by targeting the cancer cell surface in parallel to promoting carcinogenesis. Studies in ovarian cancer have determined the role of LL-37 in recruiting multipotent mesenchymal stromal cells (MSCs), which have the capacity to produce growth factors and cytokines [110, 111]. Experiments in nude mice revealed that the development of blood channels and LL-37 promoted angiogenesis. CAMP was significantly increased in breast cancer cells as a result of signaling through ErbB2. CAMP in breast cancer cells also induces the production of cytokines through interacting macrophages [112]. It behaves as a growth factor for lung cancer via the activation of epidermal growth factor receptor (EGFR) [113].

Devil facial tumor disease (DFTD), a contagious cancer, caused the Tasmanian devil (*Sarcophilus harrisii*) to slip into the endangered list. The ACP cathelicidins isolated from DFTD cells exhibited potent anticancer and inflammatory effects [114]. Gene Ontology (GO) and Ingenuity Pathway Analyses of the mature cathelicidin peptides Sarcophilus harrisii-cathelicidin 1 (Saha-CATH1), Saha-CATH2, Saha-CATH3, Saha-CATH4, Saha-CATH5, Saha-CATH6 and Saha-CATH7 revealed the downregulation of genes involved in DNA replication, cell cycle progression and checkpoints. Furthermore, it induces endoplasmic reticulum stress and downregulates protein hydroxylation. A cytotoxicity assay revealed that CATH5 is cytotoxic and reduces cell viability at concentrations equal to or greater than 125 µg/mL [115].

The hemolytic activity of the avian cathelicidin ortholog from ducks in the dCATH assay against anticoagulated human blood and in the cytotoxic assay [3-[4,5-dimethylthiazol-2-yl]-2,5 diphenyl tetrazolium bromide (MTT) assay] using HaCat cells indicated 50% lysis of mammalian erythrocytes at 20 μM and a very low survival rate of HaCat cells supplemented with a high concentration of dCATH in the medium. An assay involving the use of the fluorescent dye *N*-phenyl-1-napthylamine (NPN) to evaluate the permeability of the lipid membrane to the NPN demonstrated that, in a dose-dependent manner, dCATH permeabilized the membrane even at concentrations lower than the evaluated minimum inhibitory concentration (MIC) for antimicrobial activity [116].

An *in vivo* study on the effects of chicken cathelicidin on Ehrlich ascites cells (EAC) revealed a 90–95% suppression of cell line growth after exposure to 40 µg/mL chicken cathelicidin for 72 h. Subcutaneous implantation in a mouse tumor model validated the effect of cathelicidin on the downregulation of the cyclin A1 and cyclin D genes in MCF-7 cells [117].

Since cathelicidin can target cancer cells while bypassing healthy cells, it can eliminate cancerous cells without the complications associated with many other cancer treatments, such as chemotherapy and radiotherapy. It can reinforce the immune system and promote the hope of preparing cancer therapeutics, which is critical in fighting cancer. The role of cathelicidin in preventing cancer cells from migrating promotes a better treatment response through the enhancement of immunity in the body [118].

### Magainin

Magainins were first discovered in the skin of *Xenopus laevis* by Michael Zasloff at the National Institutes of Health (NIH) and Dudley H Williams at the University of Cambridge [119]. It works mainly on the lipid matrix of the membranes. Magainin is cationic in nature and permeabilizes the cell membranes of the bacterial community [120]. In one study, magainin II was demonstrated to induce cell death via membrane pore formation. Magainins have been found to affect cancer cells but not normal cells [68]. It has shown cytotoxicity against various human cell lines (human). It has the potential to be used as an immunomodulatory agent and was considered an emerging antibiotic in 1998. These peptides were found to lyse different murine and human cancer cells [121].

The antitumor activity of magainin was tested against lung cancer cell lines by Ohsaki et al. [122], who reported that exposure to magainin inhibited the cancer cell proliferation. The tested magainins (MAG A and B) showed better antiproliferative response in combination with standard chemotherapeutic drug cisplatin (combined IC_50_ doses of magainin and cisplatin) and reduced the percentage of surviving lung cancer cells by 29% [122]. The antiproliferative and cytotoxic effects of magainin were also examined by colorimetric Wild Type Staining-1 (WST-1), bromodeoxyuridine (BrdU), and lactic dehydrogenase (LDH) assays in three bladder cancer cell lines (RT4, 647 V, and 486 P) and in a murine fibroblast line. The peptide was found to exert selective antitumor activity in bladder cancer cell lines (RT4, 647 V, and 486 P), and no effective activity was observed in murine fibroblast lines or human fibroblasts. The induction of cell death is accomplished by promoting pore or channel formation in the bladder cell membrane [121]. One of the progressive magainin peptide MSI-78 is in phase IIb/III of clinical trials for impetigo. This 22-residue magainin analog has been suggested to exhibit anticancer properties in preclinical studies [123]. A study by Liu et al. [124] (2011) showed the increased cytotoxicity of magainin upon conjugation with another peptide bombesin and suggested its applicability in targeted drug delivery. The tumoricidal activity of magainin is reported to be dose dependent as the tumor cytotoxicity against MDA-MB-231 increased with increased concentration [125]. A study aiming to understand the interaction of magainin with human cells reported colocalization of magainin with gangliosides in HeLa cells. The observed interaction was stronger as compared to interaction with typical bacterial surface phosphatidylglycerol [126].

The discussed studies suggest anticancer activity of magainin which could be enhanced by combination formulation or optimizing different delivery strategies. Yang et al. [109] (2014) used polydiacetylene micelles to deliver magainin-II and reported improved cytotoxicity both in *in vitro* and *in vivo* model systems (A549 cells and mice respectively).

### Cecropins

Cecropin is an antibacterial peptide isolated from *Hyalophora cecropia* that was first characterized in 1980 [127]. Since then, a wide variety of cecropins have been identified, and many of these molecules possess anticancer activity. Cecropins preferentially target cancer cells, triggering apoptosis or programmed cell death. These peptides are produced by insects and other invertebrates and are a part of their innate immune system [128].

Cecropins target cancer cells by programmed cell death. Cecropin XJ has been shown to prevent hepatocellular carcinoma by inducing cell cycle arrest and apoptosis after 24 h of incubation. Further, incubation with cecropin XJ induced apoptosis was suggested by dose-dependent cleavage of caspase-3 and PARP, downregulation of anti-apoptotic protein Bcl-2 and upregulation of the proapoptotic proteins Bad and BAX upon treatment with cecropin XJ [128]. The other cecropin, cecropin B also induced apoptosis and focal disruption of tumor in *in vivo* rat model of DMBA [7,12-dimethylbenz(a)anthracene] induced breast cancer [129]. Another study showed the anticancer effects of CB, CB1 (cecropin B1) and CB2 on HL-60 cells [130]. These cecropins exhibit diverse anticancer activities either by interfering with the integrity of the cancer cell membrane or by triggering different signaling pathways in the cell, resulting in apoptosis and cell death [131].

In one study, cecropin A suppressed the growth of melanomas. The phosphorylation of ERK/mitogen-activated protein kinase (MEK), which controls cell proliferation, has been demonstrated to be decreased by cecropin A [132]. A study conducted by Pascariu et al. [133] used four adherent cell lines—MDA-MB-231 (disease: mammary adenocarcinoma; morphology: epithelial), HT-29 (disease: colorectal adenocarcinoma; morphology: epithelial), A549 (disease: human alveolar carcinoma; morphology: epithelial) and M14K (disease: human metastasis; morphology: mesothelial) to study the effects of cecropin A and B [133]. Cell viability was evaluated using the MTT assay which showed 7% and 10% cytostasis for cecropin A and B, respectively, at lower concentrations. Higher concentrations of these molecules resulted in a greater cell death rate.

A study by Wu et al. [134] examined the activity of cecropin XJ both *in vivo* and *in vitro* against normal gastric epithelial GES-1 cells and human gastric cancer BGC823 cells. The results of the *in vitro* MTT and colony formation assays indicated that cecropin XJ suppressed the proliferation and colony formation of BGC823 cells but had no inhibitory effect on normal gastric epithelial GES-1 cells. *In vivo* studies were carried out with BGC823 cell-induced mouse models. Cecropin XJ induced cancer cell apoptosis and inhibited angiogenesis. Xu et al. [78] investigated the anticancer activity of *Bombyx mori* cecropin A and cecropin D against human esophageal cancer Eca109 and TE13 cells and normal human embryonic kidney 293T cells. The colony formation assay and cell counting kit-8 results indicated a progressive reduction in cell proliferation and reduced colony formation in both cancer cell lines but no restriction of normal human embryonic kidney 293T cells.

These findings suggest that cecropins may possess broad-spectrum anticancer activity, as they may function effectively against several types of cancer. Cecropins have shown promising results in preclinical testing, but they have not yet been tested as anticancer medications in clinical investigations. However, the development of peptide-based therapeutics is limited, which could explain why they are not easily accessible. Nevertheless, recent improvements in the synthesis and delivery of peptides have allowed them to be used in some applications. Thus, it is possible that clinical studies examining the use of cecropins for anticancer purposes may take place soon. The mode of action of cecropins must be fully understood which would help to enhance their therapeutic potential through further studies and trials. However, since they are unique, cecropins are promising candidates for novel cancer treatments.

## Mechanism of action

ACP-based therapy is gaining popularity in field of cancer treatment and research and usually involves targeting tumor cells at three different stages: initiation, growth and development. These peptides have great potential compared to other anticancer treatments due to their specific targeting properties and fewer side effects. Usually, tumors create a suppressive environment that hinders immune response and ACPs can reprogram the TME. M2-like TAMs promote tumor growth and these peptides inhibit their recruitment proving itself as a promising therapeutic strategy [135]. There are several modes of action of these peptides that help to prevent tumor cell growth, proliferation and spread.

### Inhibition of DNA synthesis and replication

A peptide obtained from the hemolymph of the insect *Heliothis virescens* was tested for its antitumor properties by the National Cancer Institute (NCI) against a variety of cancer cell lines, and it was reported to be effective against 60% of the cancer cell lines (34 out of 56). The results showed that this peptide led to targeted inhibition of DNA synthesis and replication in cancer cells, which caused growth inhibition (by approximately 50%), hence hindering tumor progression with minimal toxicity to normal cells. The peptide was also found to be effective against the cancer-promoting Epstein-Barr virus [136].

ACPs binds to specific structures (Holliday Junction Intermediates) in DNA which are involved in replication and repair and prevent the enzymes from fixing DNA breaks. The damage then causes cell response halting cell division and resulting in cell death. Two peptides wrwycr and wrwyrggrywrw were tested on different cell lines such as PC3, Du145, etc. MTT assay revealed that all these cell lines were quite sensitive to the treatment and showed reduced survival rates with 50–100 µM dose of wrwycr and 10–50 µM dose of wrwyrggrywrw [137].

### Angiogenesis inhibition

Tumor cells can express vascular endothelial growth factor (VEGF), which is responsible for the formation of new blood vessels. Some ACPs (such as KV11) act as anticancer agents by inhibiting blood vessel formation in human umbilical vein endothelial microvascular endothelial cells (HUVECs) with an IC_50_ value of 15 µM without causing harm to the normal cells. In addition, ACPs also enter cells, causing damage to organelles, specifically mitochondria. This leads to disruption of the normal functioning of cells, which results in apoptosis or programmed cell death [138].

### Immune regulators

ACPs can also act as immune regulators by regulating cells (such as CD4^+^, CD8^+^, and NK cells) that are involved in generating a strong immune response. LfcinB is a peptide obtained from cow’s milk that works by increasing cytokine production, which is essential for activating the immune system. Wolf and his colleagues [139] reported that lactoferricin can help to restore the depletion of CD4^+^ and CD8^+^ cells in mice treated with cyclophosphamide. This restoration leads to the generation of a strong immune response. Several ACPs have different mechanisms by which they can either directly kill cancer cells or enhance the immune system to fight against tumors, suggesting their potential to be utilized as an option for anticancer treatment [136].

### Membrane disruption and apoptosis

The membranes of healthy cells and cancer cells differ from each other and this difference plays a key role in how ACPs specifically target cancer cells. Cancer cells have a more negative charge than the healthy cells due to molecules like phospholipids and sialic acid, that attracts ACPs. Also, the membrane fluidity of cancer cells is more than healthy ones, which makes them more susceptible to ACPs that can destabilize the membrane leading to death. Apart from this, cancer cells have more surface area because of more microvilli, which provides more attachment target sites for ACPs [139].

There are many ACPs (around 818) which show their effect on the cancer cells via apoptosis and they are stored in a database called “Apoptosis-Inducing Anti-cancer Peptide Database” [140]. Some ACPs such as RA-V are capable of disrupting mitochondrial membrane leading to the release of cytochrome c, which subsequently leads to apoptosis [62]. Apoptosis is regulated by Bcl-2 proteins, and certain peptides are being developed to imitate or strengthen the pro-apoptotic properties of Bcl-2 family members [141].

**Figure 4:**
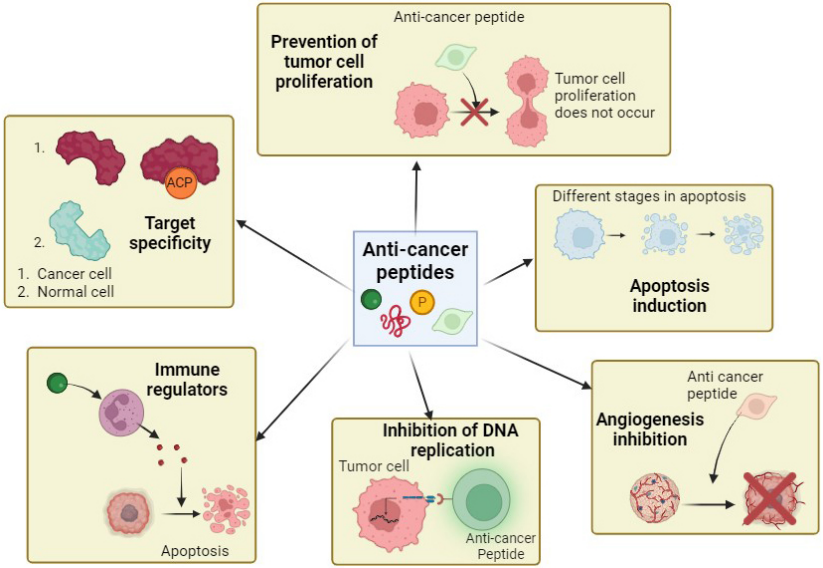
Mechanisms of action of anti-cancer peptides. These peptides exhibit different ways of action that leads to anti-cancer properties. The anti-cancer peptides may lead to apoptosis induction, immunomodulation, angiogenesis inhibition, etc. Different peptides can exhibit one or a combination of these mechanisms. Created with BioRender

Some ACPs like Mellitin, directly disrupt the cell membrane while some, like ChMAP-28, cause necrosis, leading to cell death [139]. For example, the antitumor effect of LL-37, a cathelicidin peptide, has been demonstrated to cause membranolytic death. Similarly, these peptides are effective antitumor agents and can also modulate the immune system. This helps them recognize and destroy cancer cells. Figure 4 shows the different mechanisms of action of ACPs.

### Anti-metastatic mechanism

According to research done by Yurong et al. [142], an aptamer has been created to target CKAP4, a protein that is implicated in the motility and adherence of cancer cells. It stops cancer cells from sticking to surrounding tissue. Cell motility is dependent on the PI3K/AKT signalling pathway, which is disrupted by it. The aptamer stops cancer cells from moving and sticking to one another, which stops metastasis (the spread of cancer cells throughout the body) [142].

## Other uses of ACPs

Initially, researchers discovered that antimicrobial peptides that act as ACPs have the potential to inhibit bacteria, fungi, and viruses [143]. These agents have the ability to interrupt microbial membranes and hence can be candidates for treating various infectious diseases, including infections resistant to antibiotics [144].

Numerous studies have examined the ability of numerous ACPs, such as defensins, to inhibit viral entry, replication, and assembly. These are antiviral medicines that can be used for treating many viral diseases. Some major drugs include remdesivir and chloroquine. Both of these factors inhibit the replication of viruses [145]. Perishable goods can be made available for longer periods by employing ACPs to minimize the growth of deteriorating microorganisms such as bacteria and other pathogens. Many ACPs also have immunomodulatory effects because of increased cytokine production and increased immune cell activity [146, 147].

The coupling of ACPs to nanoparticles helps improve drug delivery to specific target sites, such as cancerous tissues [148]. This methodology increases the uptake of the drug and its therapeutic efficacy and stability. Several ACPs contribute to tissue repair and wound healing through accelerated regeneration of tissues, control of inflammation, and promotion of angiogenesis [149, 150]. With these qualities, they can be employed in making topical formulations and wound dressings for the management of chronic wounds.

Biofilms are a mass of microbes that are resistant to antibiotics. Since these peptides can penetrate and degrade biofilms, ACPs are suitable for the prevention and treatment of biofilm-related diseases [151]. Additionally, some ACPs have adjuvant properties that enhance the immune response to vaccines. Vaccines can be made more effective for immunization against cancer and infectious diseases [152]. Hence, not only anticancer agents but also antimicrobial peptides play significant roles in other areas.

## ACPs associated challenges and approaches to overcome those challenges

Though, ACPs offer several advantages over other therapies, such as affordability, high effectiveness, lower toxicity, high tolerance, their development is a major challenge owing to its time-consuming designing, production, and higher cost. Another major challenge is their short half-life and proteolytic cleavage resulting in quick elimination from the body requiring frequent doses [153].

These limitations and challenges warrant more mechanistic studies to be conducted to bring these peptides into translational products for cancer treatment. Literature suggests mixing of these microbial peptides with other therapies, such as chemotherapy or immunotherapy to overcome these limitations, which can augment their anticancer effects and increase patient survival rates [154]. Understanding how the cells function under the influence of TME can be quite helpful to develop better combination therapies [155].

Further, to overcome the challenges of designing these peptides at a faster pace, different AI approaches such as deep learning (DL), machine leaning (ML) as well as hybrid learning (a combination of both ML and DL) can be used. It offers a more economical way to identify and separate ACPs from non-ACPs and then categorizing them based on their effectiveness [156]. To improve the half-life of such peptides, they can be conjugated with transport proteins such as transthyretin exerting protection from degradation. Apart from transthyretin, PEG or XTEN can also be used to improve half-life of ACPs. To overcome the issues of cell permeability, researchers attach CPPs (cellular delivery tags) to these peptides that help them enter cancer cell membrane easily [157]. immune checkpoint inhibitors (ICIs), that target PD-1 and CTLA-4, have recently shown progress in clinical trials. This gave rise to a new class of anti-cancer medications and in the field of immuno-oncology, however, these inhibitors are also associated with side effects like toxicity, need for intravenous administration and limited efficacy. The combination therapy of ICIs with ACPs is being explored to obtain better outcomes [23]. ACPs blocking immune checkpoints via surface interaction with checkpoint proteins, also referred as peptide inhibitors are suggested to activate T cell against tumor cells after releasing immune breaks [158]. Such inhibitor peptides, destabilizing the protein-protein interactions at immune checkpoints are designed using rational designing from known structures, empirical designing from sequence data and computational technology followed by phage display [23]. A recent study reported the designing and development of a helix-loop-helix peptide ERY 2-4 exhibiting the selective inhibition of immune checkpoint CTLA4-B7 and activating T cells [159].

Thus, the different combination therapies, carrier mediated delivery, rational designing of the peptides, combination of ACP and ICI approach as one are some of the approaches to deal with the challenges associated with ACP mediated cancer therapy.

## Clinical trials and future trends

Several anti-cancer peptides are currently under clinical trials or in the pre-clinical stage. Over the last decade, an increasing number of anti-cancer medications have been brought to the market for clinical studies. A gonadotropin-producing hormone analog, Lupron, had worldwide sales of over two hundred million dollars in the year 2011. There are 29 peptide-based drugs as in drug bank database out of which 5 have been approved and 11 are still under trials (Table 2). The approved peptides on tumour cells inhibit cancer growth by targeting anti-cancer immune response, hormone signalling, cancer cell death, and cellular transcription [140].

**Table 2:** Peptide based anti-cancer drugs in Drug Bank Database [160]

**Category**	**Number of drugs**	**Names of drugs**	**Effective against**
Approved	5	Plitidepsin	Multiple myeloma
		Dactinomycin	Different cancers
		Triptorelin	Prostate cancer
		Tebentafusp	Uveal melanoma
		Buserelin	Breast cancer
Investigated	13	Lobradimil	Brain and CNS tumors
		Tigapotide	Prostate cancer
		SF1126	Neuroblastoma
		Iseganan	Head and neck cancer
		G17DT	Colorectal cancer
		IRL-1620	Brain and spinal cord issues
		CTCE-0214	Various cancers
		ATN-161	Carcinoma
		Teverelix	Prostatic adenoma
		Nelipepimut-S	Breast cancer
		Canfosfamide	Fallopian tube/Ovarian cancer
		PM02734	Advanced malignant solid tumors
		Darinaparsin	Advanced solid tumors
Trial	11	TAK-448	Prostate cancer
		Ozarelix	Prostate cancer
		LTX-315	Soft tissue sarcoma
		VEGFR2-169	Pancreatic cancer
		Bombesin	Prostate carcinoma
		Valspodar	Breast cancer
		Dolastatin 10	Pancreatic cancer
		Soblidotin	Sarcoma
		Zoptarelin doxorubicin	Endometrial cancer
		Balixafortide	Locally recurrent breast cancer
		Bleomycin A6	Squamous cell lung cancer

The first FDA-approved anti-cancer peptide, Bortezomib, demonstrates the potential this technique holds for cancer treatment. The knowledge about their mode of action and the clinical applications of anticancer microbial peptides is constantly expanding. These peptides could be used as effective weapons for combating cancer due to the increasing need for new cancer therapeutics. By using various modified amino-acids as well as other conjugation techniques, their stability can be enhanced leading to better pharmacokinetics. Scientists are also working towards personalized ACP therapies based on the characteristics of different cancer patients [153].

ACPs have shown revolutionary results as major therapeutics against different cancer types and with advances in computational methods and proper understanding of their structures and activities can lead to greater theranostic success [161].

## Conclusions

The convergence of innate immunity and oncology points to a positive direction within which ACPs show great potential as immune modulators in the fight against cancer. ACPs act as several immunological modulators by modulating T-cell activation, inducing apoptosis, helping in membrane lysis, etc. These functions suggest the exploration of ACPs during the rising demand for novel cancer therapeutics. Further demonstrated success when used in combination with established treatments and immunotherapies also suggest the need to explore ACP based treatment approaches in detail. In combination with currently available treatments, the immunomodulatory activity of ACPs can reactivate dampened immune responses against tumors. This leads to an increase in immunity, which further helps in fighting cancer.

During the transition from preclinical studies to clinical studies of such combinations, various issues need to be thoughtfully considered. Some of the major issues include dose, formulation of proper combinations, and patient selection criteria. Similarly, investigations of any possible side effects and long-term consequences of ACP therapy have become necessary. One promising line of research relates to personalized treatment by means of ACPs that may be altered according to a specific patient’s immunogenomic profile.

This review emphasizes the evolutionary potential of ACPs as immunomodulatory drugs. In the ever-changing arena of anticancer therapies, these ACPs are on the verge of creating new grounds for a large discovery.

## Abbreviations

ACPs: anticancer peptides
BrdU: bromodeoxyuridine
CAR: chimeric antigen receptor
cFLIP: cellular FLICE-inhibitory protein
COP1: cytosolic dual-specificity phosphatase 1
DFTD: Devil facial tumor disease
EAC: Ehrlich ascites cells
EGFR: epidermal growth factor receptor
ERK: extracellular signal-regulated kinase
GLOBOCAN: Global Cancer Observatory
GO: Gene Ontology
HER2: human epidermal growth factor receptor 2
HLAs: human leukocyte antigens
HNP-1 to 3: human neutrophil defensins 1-3
HUVECs: human umbilical vein endothelial microvascular endothelial cells
IAP: inhibitor of apoptosis
ICIs: immune checkpoint inhibitors
IL-2: interleukin 2
LDH: lactic dehydrogenase
MDSCs: myeloid-derived suppressor cells
MIC: minimum inhibitory concentration
MSCs: mesenchymal stromal cells
MTT: 3-[4,5-dimethylthiazol-2-yl]-2,5 diphenyl tetrazolium bromide
NCI: National Cancer Institute
NHL: non-Hodgkin lymphoma
NK: natural killer
NPN: *N*-phenyl-1-napthylamine
PS: phosphatidylserine
Saha-CATH1: Sarcophilus harrisii-cathelicidin 1
TAAs: tumor-associated cells
TAMs: tumor-associated macrophages
TANs: tumor-associated neutrophils
TME: tumor microenvironment
Tregs: regulatory T cells
VEGFR2: vascular endothelial growth factor receptor 2
WHO: World Health Organization
WST-1: Wild Type Staining-1

## Declarations

### Copyright

© The Author(s) 2024.
